# Challenges and adaptation strategies for Riesling grape (*Vitis vinifera* L) production in the southwest desert in the USA

**DOI:** 10.3389/fpls.2025.1621299

**Published:** 2025-09-17

**Authors:** Most Tahera Naznin, Md Obyedul Kalam Azad, Jill Moe

**Affiliations:** ^1^ Department of Agriculture, Veterinary and Rangeland Sciences, Nevada Agricultural Experiment Station, University of Nevada, Reno, Las Vegas, NV, United States; ^2^ Desert Farming Initiative, University of Nevada, Reno, NV, United States

**Keywords:** Riesling cultivation, desert viticulture, water management, climate adaptation, precision agriculture

## Abstract

Riesling, a traditionally cool-climate grape variety, faces increasing challenges when cultivated in the Desert Southwest region of the United States, where extreme heat, water scarcity, and nutrient-poor soils prevail. This paper reviews published research data to identify the key stress factors affecting Riesling production in these arid environments and explores adaptation strategies to enhance its viability. This paper synthesizes potential strategies for adapting Riesling cultivation to desert environments under increasing climate variability. These strategies include high-elevation planting, the use of drought-tolerant rootstocks (e.g., Ramsey, 1616C, 110R, 1103P, 140Ru), and canopy or trellising systems to mitigate thermal stress and optimize microclimates. Soil health improvements through mulching, composting, cover cropping, and biochar amendments are also reviewed for their roles in enhancing water retention and nutrient cycling. Smart irrigation technologies utilizing IoT-based soil moisture sensors and AI-driven scheduling are discussed as tools for maximizing water efficiency. Additional innovations, such as climate modeling, remote sensing for vineyard management, and agrivoltaic systems, are explored for their potential contributions to sustainable vineyard design and operation. Through this literature review, it appears that Riesling production could adapt to desert climates by integrating traditional practices with precision agriculture and sustainability-driven innovations. While these strategies show promise in supporting fruit quality and long-term resilience, however, further applied research is needed to validate their effectiveness in specific arid contexts.

## 1 Introduction

**Figure 1:**
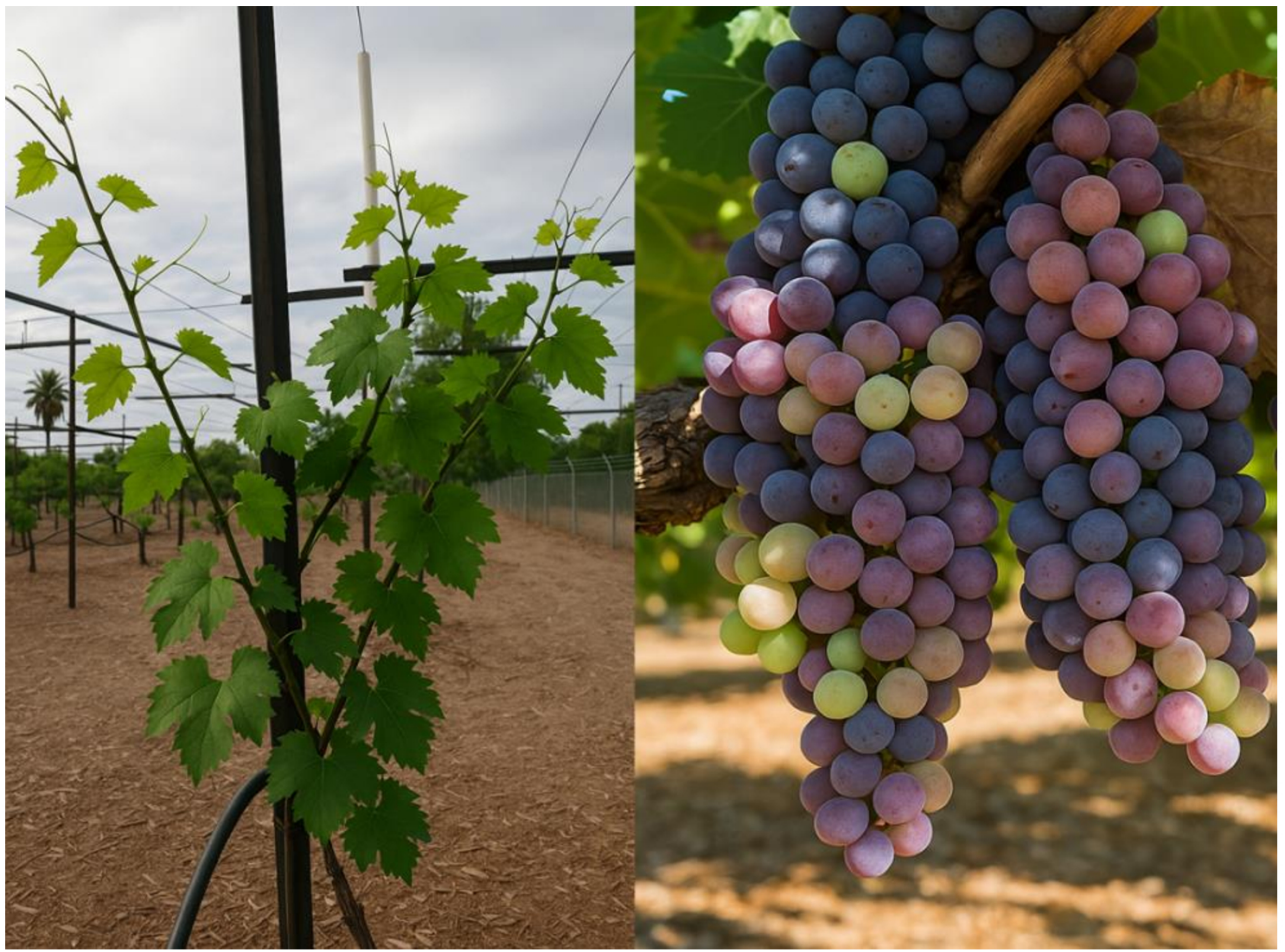
Riesling plant (left) and ripened grapefruit (right).

Grapevine cultivation is significantly influenced by environmental conditions, soil type, and climate, all of which determine the vine growth characteristics and quality of the fruit (Meneghelli et al., 2018; Irimia et al., 2015). Originally from Germany, Riesling (*Vitis vinifera* var. *Riesling*) is a cool-climate grape and sensitive to environmental conditions (Li et al., 2019). It produces moderate yields with early bud break and mid- to late-season ripening, making it vulnerable to spring frosts and heat stress (Keller, 2020). Oenologically, Riesling is prized for its floral, citrus, and stone fruit aromas, with wines ranging from dry to sweet and showing excellent aging potential due to their acidity and balance (Robinson et al., 2013). **Figure 1** illustrates two key stages of grapevine development: the left shows a young vine in the vegetative stage with active shoot and leaf growth, while the right displays mature clusters at veraison, marking the onset of ripening. This highlights the vine’s phenological progression from early growth to fruit maturity.

Globally, Riesling thrives in Germany (Mosel, Rheingau), France (Alsace), Austria (Wachau), Australia (Clare and Eden Valleys), and New Zealand (Marlborough). In the U.S., key Riesling-producing areas include Washington State, New York’s Finger Lakes, Oregon, Michigan, and select cooler sites in California and Idaho (UC Davis Viticulture and Enology, 2023, University of California, Davis, 2023). These regions provide ideal growing conditions, long growing seasons, and well-drained soils that allow grapevines to develop their signature balance of acidity, minerality, and fruit-forward flavors (Jones and Hellman, 2003). In the climate of Desert Southwest USA, accelerate sugar accumulation, reduce acidity, and impair flavor development. Intense sunlight also increases the risk of sunburn on exposed berries, leading to skin damage and loss of aroma and balance in the wine (Gambetta et al., 2021).

Elevated temperatures can also accelerate sugar accumulation, resulting in a shorter growing season with higher alcohol content in juice but lower aromatic complexity (Biasi et al., 2019). Another major issue is water limitation and drought stress. In the Desert Southwest USA, annual rainfall is typically less than 150 mm (6 inches), with the majority occurring during short monsoonal bursts in late summer (July–September) and occasional winter storms (December–February) (NOAA National Centers for Environmental Information, 2021). This minimal and erratic precipitation necessitates full reliance on irrigation for viticulture. However, water scarcity, allocation limits, and increasing regulatory restrictions present major challenges to the sustainability of grape production in the region (Williams and Araujo, 2002). Maintaining Riesling’s signature acidity and aromatic profile in such a climate is another key challenge (Daccak et al., 2021).

**Figure 2:**
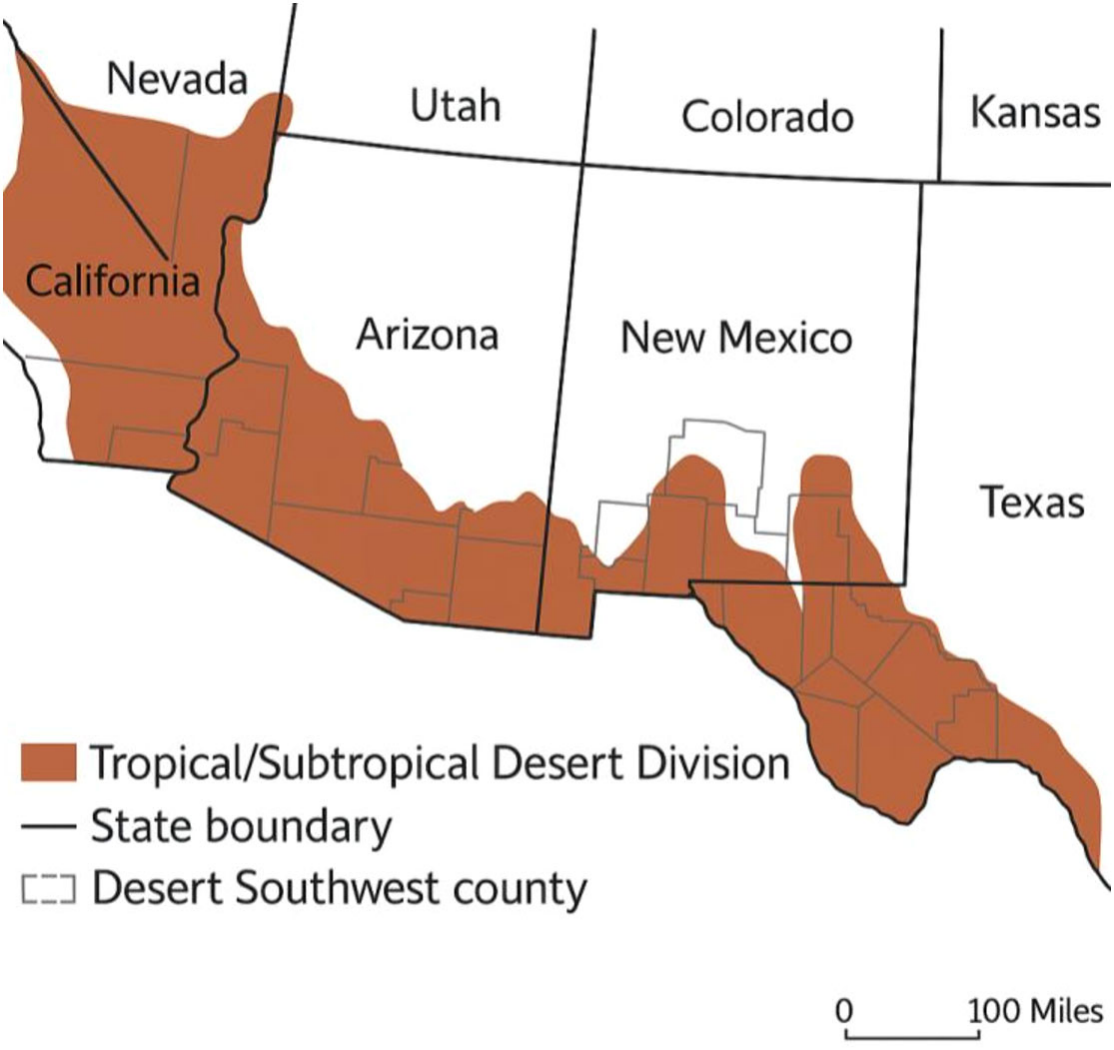
Ecological classification of the desert southwest, USA.

The southwestern regions of the United States, including Arizona, New Mexico, Texas, Nevada, and parts of California, lie along the 37^th^ parallel north (**Figure 2**). This latitude experiences moderate temperatures (25–35 °C) and ample solar radiation (2,200 kWh/m²) throughout the year (Dong et al., 2022). However, the Southwest is distinctively classified as a desert, primarily encompassing the Mojave and Sonoran deserts (Brazel, 1989). The Southwest experiences low annual precipitation, clear skies, and a warm climate year-round due to a quasi-permanent subtropical high-pressure ridge (**Table 1**). This region has an arid to semi-arid climate, characterized by high summer temperatures, minimal rainfall, frequent droughts, and significant water scarcity challenges (Azad et al., 2024). Intense heat accelerates water evaporation, increasing the demand for cooling and irrigation, while diminishing water sources like the Colorado River and groundwater struggle to support urban and agricultural needs. Prolonged droughts and excessive water consumption further stress resources, highlighting the urgency of water conservation and sustainable agricultural practices for the regions (Sanderson et al., 2015; Hirt et al., 2017).

**Table 1:** Comprehensive key weather and climate parameters of the southwest desert of the USA.

Climate parameter	Description	Typical range/Values	Reference
Temperature	High daytime temperatures	Summer: 100–120°F Winter: 40–70°F	(NWS, 2020)
Precipitation	Low annual rainfall	3–12 inches	(NOAA, 2021a, b)
Humidity	Low relative humidity	10–30%	(Arizona State Climate Office, 2019)
Wind Speed	Moderate to intense winds	5–25 mph	(NOAA, 2021a, b)
Storms	Thunderstorms, dust storms	Mostly in summer monsoon (July–Sept)	(Adams and Comrie, 1997)
Sunlight	High solar radiation	85–95% sunny days per year	(WRCC, 2020)
Elevation Effects	Temperature and precipitation vary with altitude	Higher elevations are cooler, wetter	(Barry, 2008)
Evaporation Rate	High due to intense heat and low humidity	70–150 inches/year	(Farnsworth et al., 1982)
Soil Moisture	Exceptionally low	Rapid drying after rainfall	(Scanlon et al., 2005)
Growing Season	Long, but limited by heat and water availability	250–300 days	(Winkler et al., 1974)
Snowfall	Rare in lower elevations, but occurs in higher altitudes	0–20 inches in mountains	(NOAA, 2021a, b)
Dew Point	Low most of the year	Below 30°F, higher in summer	(Arizona State Climate Office, 2020)
Climate Type	Hot desert	Arid conditions year-round	(Peel et al., 2007)

In this review, we explore the challenges and adaptation strategies for cultivating Riesling in the Desert Southwest USA. Riesling was selected for study due to its high sensitivity to climate and site-specific conditions, making it an ideal indicator for assessing the impact of extreme environments on grapevine performance. Although not the most widely planted white grape in the U.S., Riesling holds significant importance in cooler regions such as Washington State—where it is the second most planted white variety after Chardonnay—and in New York’s Finger Lakes, where it dominates white wine production (USDA-NASS, 2021; Wine Institute, 2023).

Riesling, cultivated on approximately 51,000 hectares globally, is primarily concentrated in Germany, which accounts for over 45% of its total area (OIV, 2022). Despite its prominence in cool-climate regions, its expansion into arid environments remains largely unexplored. What makes Riesling particularly significant is its sensitivity to environmental conditions, especially in relation to canopy architecture, irrigation precision, and phenological timing. These traits make it an ideal model for testing adaptive viticultural practices under climate stress. Given the increasing challenges posed by heat, drought, and extreme weather in traditional and emerging wine regions, examining Riesling’s performance in non-traditional settings such as the U.S. Southwest offers a timely opportunity to evaluate climate-resilient viticulture. This review contributes novel insights by using a well-known yet climatically sensitive variety to explore innovative growing strategies in desert viticulture, providing broader implications for global grape production under changing climates.

## 2 Optimal chemical composition of Riesling grapes

Determining the optimal chemical composition of Riesling grapes at harvest is crucial for achieving the desired sensory profile, fermentation performance, and aging potential of the resulting final products. Riesling is highly sensitive to must composition, influencing its acid-sugar balance, aromatic complexity, and typicity. While optimal ranges for Brix, pH, titratable acidity, yeast assimilable nitrogen (YAN), and aroma precursors are established in cool-climate contexts, these serve as reference points rather than fixed targets in arid regions, where heat accelerates sugar accumulation and disrupts ripening synchrony. In such environments, these benchmarks help guide adaptive practices such as irrigation management and modified harvest timing to balance physiological and compositional maturity. Using Riesling thus provides a valuable lens for assessing climate-resilient viticultural strategies (Jackson, 2020) (**Table 2**). Understanding these parameters is particularly important for Riesling, a cultivar known for its high acidity and reliance on volatile aromatic compounds especially monoterpenes like linalool, geraniol, and citronellol for varietal expression (Marais, 1983; González-Barreiro et al., 2015). Riesling’s aroma profile is also shaped by C13-norisoprenoids such as β-damascenone and TDN, which develop during ripening and contribute to aged wine complexity (Mendes-Pinto, 2009). These compounds are highly responsive to environmental conditions and harvest timing. Recent studies show that delayed harvest enhances aromatic complexity in Riesling by increasing concentrations of norisoprenoids and terpenoids (Jones et al., 2005; Previtali et al., 2022). These compounds are highly responsive to environmental conditions and harvest timing. While studies from cooler regions have shown that delayed harvest can enhance Riesling’s aromatic complexity by increasing norisoprenoid and terpenoid concentrations, such effects are often site-specific and may vary under different climatic zones and vineyard management practices (Rouxinol et al., 2023).

**Table 2:** Optimal ranges and enological parameters of Riesling grape to harvest.

Parameter	Optimal range	Purpose/Importance	Reference
Brix	20–24°Bx	Determines sugar content and potential alcohol	(Marín-Martínez et al., 2021)
pH	2.9–3.2	Enhance freshness, stability, and aging potential	(OIV, 2020)
Titratable Acidity	6.5–9.5 g/L	Contributes to balance and crisp mouthfeel	(Karime Atencia Payares et al., 2024)
Malic Acid	2–5 g/L	Retained to preserve acidity in wines without MLF	(Zoecklein et al., 1995)
Tartaric Acid	4–7 g/L	Provides stable structural acidity throughout vinification	(Sinesio et al., 2021)
Yeast Available Nitrogen	140–250 mg/L	Supports healthy fermentation, reduces risk of sluggishness	(Diverres et al., 2024)
Potassium	< 1.2 g/L	Elevated levels increase pH, potentially reducing acidity	(Schultz, 2000)
Aroma Precursors	High (terpenes, etc.)	Influence of varietal expression and aromatic complexity	(Jones et al., 2005)

These chemical markers make Riesling an ideal cultivar for studying environmental effects on grape quality. Its high sensitivity to temperature, solar radiation, and water availability allows for clear expression of terroir influences on aroma and acidity (Jones and Davis, 2000; Van Leeuwen et al., 2004). This responsiveness enables the assessment of how climate variables and adaptive viticultural strategies shape grape composition and wine typicity (Baderschneider and Winterhalter, 2000).

Furthermore, vineyard management practices such as regulated deficit irrigation (RDI) and partial rootzone drying (PRD) have been shown to modulate grape composition and wine aroma profile in Riesling, offering additional levers to optimize grape quality under changing climatic conditions (Keller, 2020). As climate change alters ripening patterns, establishing robust reference values for high-quality Riesling production becomes increasingly important. The presented data not only assists viticulturists in optimizing fruit quality but also contributes to broader enological research focused on precision harvest and sustainable production of aromatic Riesling grapes.

## 3 Factors affecting grape production

Grape production in the southwest desert region faces significant challenges due to harsh environmental conditions. Key factors such as water scarcity, climate variability, and poor soil characteristics greatly impact vine growth, fruit quality, and yield. These stressors are particularly critical in arid and semi-arid areas where viticulture depends heavily on irrigation and careful soil management. As climate change continues to alter regional weather patterns, understanding and adapting to these limitations is essential for sustainable grape production.

### 3.1 Extreme heat

The extreme heat of the southwest desert poses significant challenges to grape cultivation, particularly for Riesling. Studies have shown that temperatures exceeding ~35°C can adversely affect grapevine physiology and berry composition; however, the severity of these impacts depends on elevation, row orientation, and canopy architecture, which influence microclimate and fruit zone temperature (Greer, 2018; Cola et al., 2009).

Specifically, sustained exposure to daytime temperatures above this threshold accelerates sugar accumulation by approximately 7–14 days and can lead to a decline in titratable acidity by 1.5–2.5 g/L, depending on cultivar and site conditions (Duchêne et al., 2010; Sadras and Moran, 2012).

**Figure 3:**
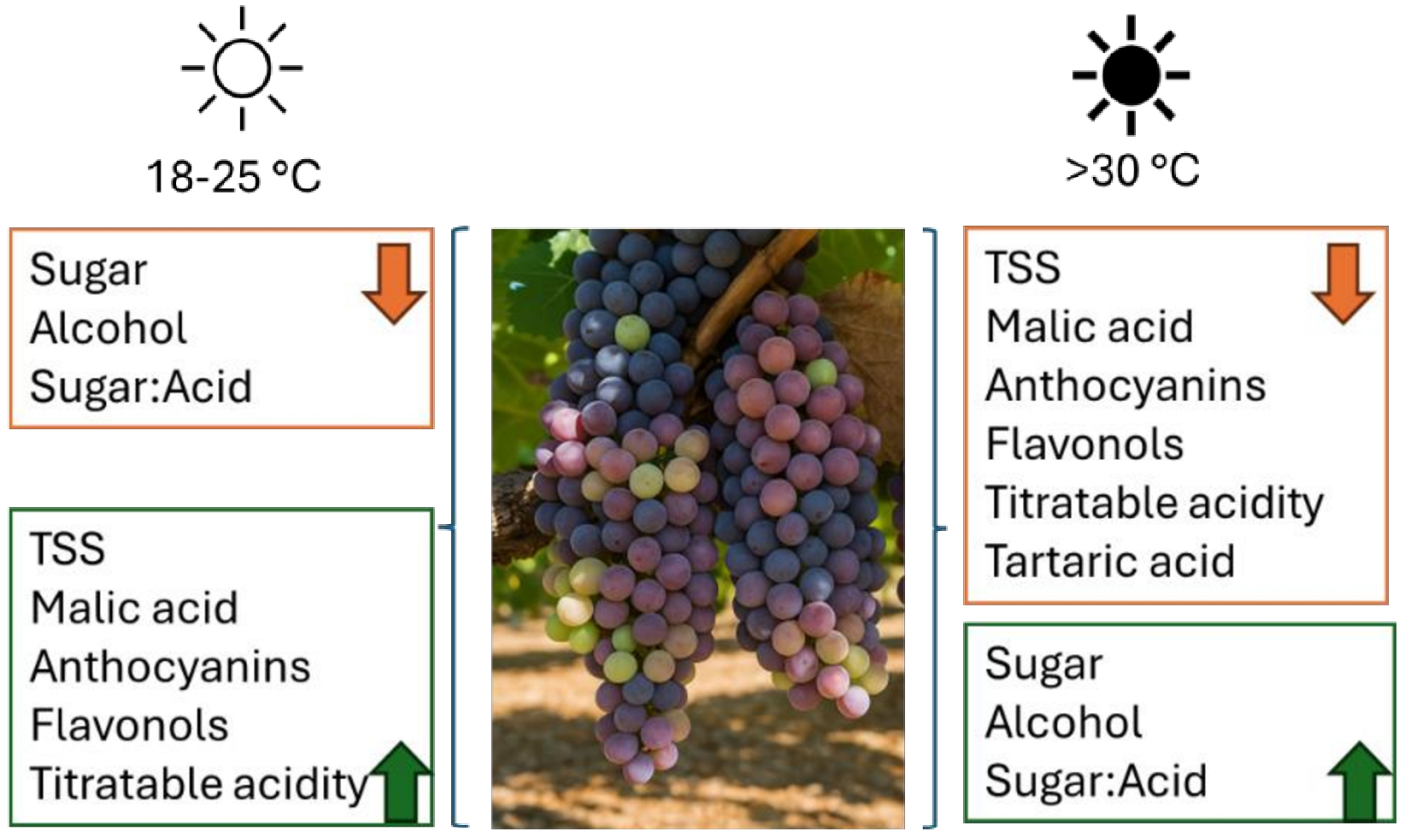
Chemical changes of Riesling grape based on temperature.

High temperatures accelerate ripening, disrupt photosynthesis, and increase sunburn risk, affecting grapefruit quality (**Figure 3**) (Abad et al., 2021; Diverres et al., 2024). Temperatures >35°C in desert vineyards accelerate ripening by increasing sugar accumulation and acid degradation, leading to a shortened harvest window and loss of balance in grape composition (Sadras and Petrie, 2011; Kizildeniz et al., 2015).

In arid regions, high diurnal temperature variation helps mitigate acidity loss by slowing malic acid degradation during cool nights, thus preserving Riesling’s acid-sugar balance under rapid ripening conditions (Rouxinol et al., 2023). This results in reduced freshness, a term referring to the sensory perception of crisp acidity and aromatic liveliness, often linked to esters and monoterpenes (Morata et al., 2019). Heat stress accelerates sugar accumulation and acidity loss, diminishes freshness, and unbalances flavors (Diverres et al., 2024). Prolonged stress reduces energy production, stunting vine growth and berry development (Chaves et al., 2010). While excessive transpiration increases water loss, potentially leading to dehydration and yield reduction (Kang et al., 2023). Sunburn further impacts grape quality, causing skin browning, flavor degradation, and uneven ripening (Intrigliolo and Castel, 2010).

Sunburn in Riesling berries typically occurs above 45°C surface temperature, but sensitivity varies. Thin-skinned Riesling is more vulnerable, and canopy management strongly influences risk leaf removal raises exposure, while shading reduces berry temperature and sunburn incidence (Greer, 2018). It causes skin browning, necrosis, and oxidation of phenolics, which degrade flavor, increase bitterness, and lead to uneven ripening (Gambetta et al., 2021). These effects are exacerbated by high transpiration rates, leading to dehydration and reduced yield (Medrano et al., 2015).

### 3.2 Water scarcity and drought stress

Water availability is a critical factor in viticulture in the Southwest Desert. Annual precipitation in regions such as southern Nevada and Arizona often falls below 150 mm, while reference evapotranspiration (ET_o_) can exceed 1,400–1,600 mm/year, resulting in a large water deficit (Jones et al., 2022; Keller, 2023). For grapevines, seasonal water requirements typically range between 500–700 mm, depending on canopy size, crop load, and climatic conditions (Ojeda et al., 2002). Thus, available rainfall covers less than 20–30% of the crop’s seasonal demand, making supplemental irrigation essential. Insufficient irrigation defined as less than 40–50% of ET_o_ during key phenological stages (e.g., post-veraison) can lead to impaired vine growth, reduced berry size, and lower yield and quality (Keller, 2023; Intrigliolo and Castel, 2010).

**Figure 4:**
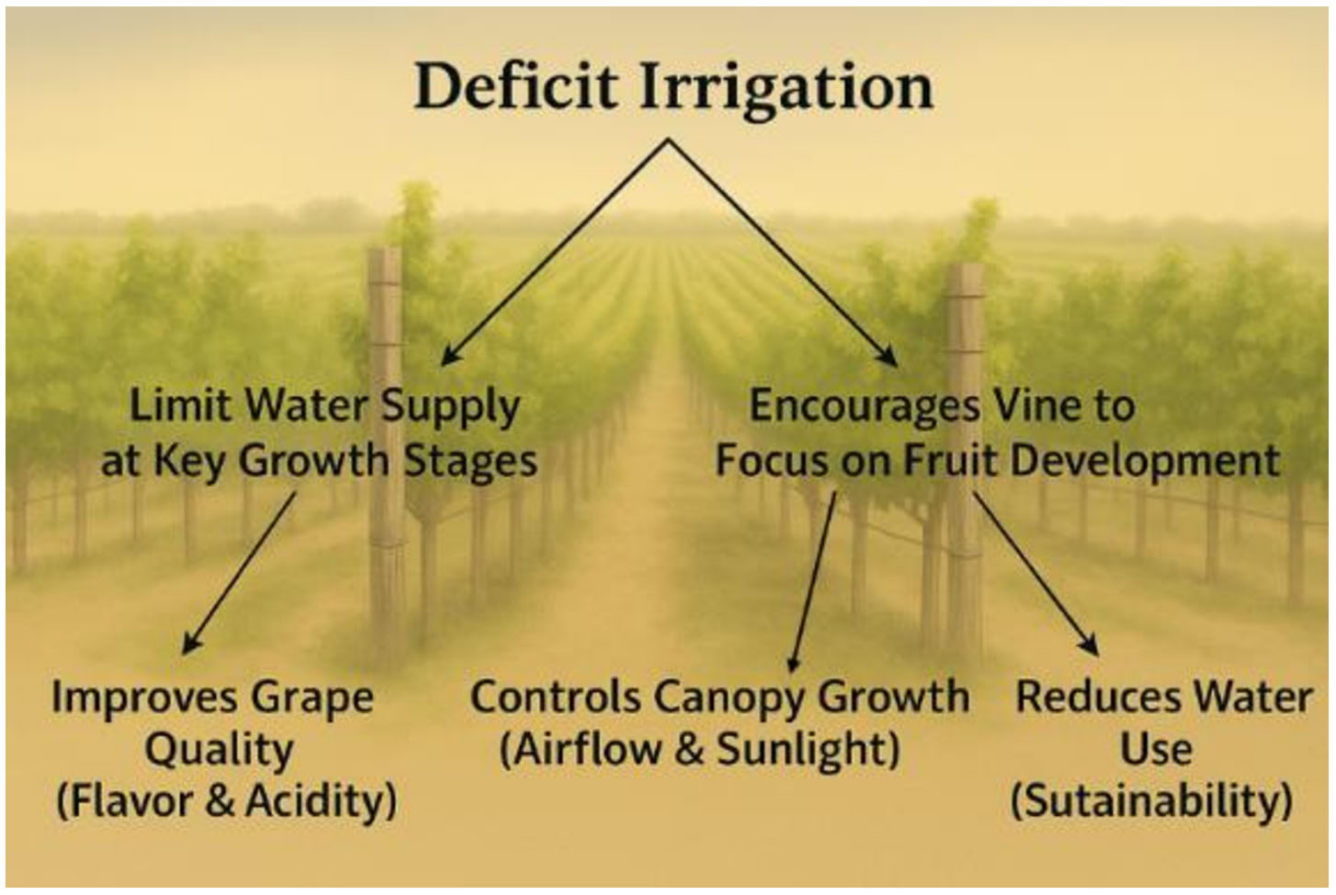
Deficit irrigation methods.

Insufficient water availability can lead to reduced vine vigor, resulting in stunted growth and diminished canopy development. This reduction in photosynthetic activity hampers the vine’s ability to produce energy, adversely affecting both vegetative growth and fruit production (USDA Agricultural Research Service, 2023). Consequently, berry size may decrease, leading to lower yields and compromised vine health. Precise water management, such as regulated deficit irrigation, can reduce berry size and increase the skin-to-juice ratio, enhancing flavor, color, and aroma concentration—making it a key strategy for optimizing grape quality in arid regions (Keller et al., 2023).To conserve water while maintaining vine health, many vineyards implement deficit irrigation strategies (Rouxinol et al., 2023) (**Figure 4**). This technique limits water supply at specific growth stages, encouraging vines to focus energy on fruit development rather than excessive vegetative growth. Properly applied, deficit irrigation can improve grape quality by concentrating flavors and maintaining acidity, control canopy vigor, and significantly reduce water consumption (Martínez-Lüscher et al., 2020; Gadoury et al., 1997).

In Riesling, moderate water stress becomes detrimental when physiological thresholds such as stem water potential drop below 1.2 MPa or stomatal conductance falls below 50 mmol m^-2^ s^-1^, leading to reduced photosynthesis, impaired ripening, and increased risk of sunburn and acid loss. These thresholds are determined through field measurements of vine water status and gas exchange, and highlight the need for precise irrigation to balance fruit quality and vine function (Schultz, 2003). Typically, 50–75% of the plant’s evapotranspiration (ET) is replaced, inducing moderate water stress that enhances berry composition without severely impacting yield (Cabral et al., 2022). Research from the University of Nevada, Reno, led by Grant Cramer, also suggests that replacing about 75% of ET optimizes grapevine performance while conserving water (https://www.unr.edu/nevada-today/news/2021/grant-cramer-retirement, 2021; UNR, 2021). Careful monitoring, such as tracking midday leaf water potential, is critical to ensuring that vines experience beneficial, controlled stress (Williams, 2010). Another effective water conservation method is drip irrigation, which delivers precise amounts of water directly to the vine roots (Reta et al., 2025). The typical irrigation system in the grapevine is presented in the table below (**Table 3**).

**Table 3:** Typical vineyard irrigation methods and water requirements.

Irrigation Method	Description	Required (L/vine/week)	Efficiency	Suitability	References
Drip Irrigation	Delivers water directly to the root zone via emitters; minimizes evaporation.	20–80	High (85–95%)	Highly recommended; precise and efficient.	USDA Natural Resources Conservation Service (NRCS), (2012)
Surface (Flood/Furrow)	Water flows over the soil surface to reach the vines.	150–250	Low (40–60%)	Not ideal due to high evaporation and runoff.	USDA Natural Resources Conservation Service (NRCS), (2012)
Sprinkler Irrigation	Overhead application through nozzles; useful for cover crops or frost control.	80–150	Moderate (60–75%)	Limited use; inefficient under hot, windy conditions.	USDA Natural Resources Conservation Service (NRCS), (2012)
Subsurface Drip (SDI)	Underground drip lines deliver water directly to root zone below the surface.	15–60	Very High (90–98%)	Excellent for water conservation and reduced weed growth.	Ale et al. (2009); USDA Natural Resources Conservation Service (NRCS), (2012)
Manual/Basin Irrigation	Water is applied manually to basins around vines.	100–200	Low–Moderate	Sometimes used in small-scale or traditional vineyards.	USDA Natural Resources Conservation Service (NRCS), (2012)

### 3.3 Spring frost

Late spring frosts pose a significant threat to sensitive grape varieties such as Riesling, often leading to poor fruit set, reduced yields, and long-term vine damage. Riesling’s early bud break—typically in March to early April in the U.S. Southwest makes it especially vulnerable to post-dormancy frost events (Gladstones, 2011). Recent climate shifts have widened this frost risk window by advancing bud phenology without reliably delaying last frost dates (Darbyshire et al., 2016). Although predictive models exist, few are tailored to desert viticulture, where rapid warming and diurnal extremes call for site-specific tools such as growing degree days (GDD) based models and cold-air drainage mapping.

Historical climate data from regions like southern Nevada and northern Arizona show spring frost events occur on average 1–3 times per decade, with last frost dates ranging from mid-March to early April, depending on elevation and microclimate (NOAA, 2023; Jones et al., 2005). Even infrequent frosts during this period can cause severe damage due to the phenological sensitivity of Riesling.

Valleys within vineyards are especially susceptible because cold, dense air naturally flows downhill and settles in low-lying areas, a phenomenon known as cold air drainage, which increases the risk of frost damage (Snyder and de Melo-Abreu, 2005). Consequently, site selection is crucial; growers often prefer planting on slopes or elevated areas with better air drainage to minimize frost exposure. Moreover, the implementation of frost protection measures such as wind machines, which mix warmer upper air with colder surface air, or overhead sprinkler systems that insulate buds through the release of latent heat during freezing, becomes essential to safeguard production in frost-prone environments (Gutiérrez-Gamboa and Mucalo, 2025). Effective frost management strategies are critical for maintaining consistent yields and fruit quality in regions cultivating sensitive varieties like Riesling.

### 3.4 Climate variability and extreme weather events

The desert regions also experience sudden temperature fluctuations and seasonal weather extremes that can significantly impact vine health, flowering, fruit set, and overall grape quality (Jones et al., 2005). One of the defining climatic features of this region is its dramatic diurnal temperature shift; daytime temperatures often soar above 38°C, while nighttime temperatures can drop sharply to 15-20°C, resulting in a daily temperature drop of 15–25°C. These cool nights are beneficial for preserving grape acidity; however, sudden drops in temperature, particularly in late spring, can result in damaging frosts (Kalantari et al., 2024). Conversely, extreme summer heatwaves impose additional stress on vines. Prolonged exposure to temperatures above 35°C can impair photosynthesis, reduce pollen viability through denaturation of key proteins involved in pollen development and germination, and lead to poor fruit set, uneven ripening, and sunburn on grape clusters (Rogiers et al., 2022). Moreover, elevated heat levels accelerate sugar accumulation, which leads to earlier harvest dates and a drop in fruit acidity, altering the balance and flavor profile of the resulting wines (Rouxinol et al., 2023).

The summer monsoon season (July to mid-September) in the Southwest brings brief but intense rainstorms and sharp rises in humidity, with levels jumping from 15–25% to 60–80% and rainfall totaling 50–150 mm during the season (Liebmann et al., 2008; NOAA, 2023). These conditions, occurring 2–3 times per week, create ideal environments for fungal diseases like powdery mildew and botrytis bunch rot. Without proper canopy management, fungicide use, and airflow, these pathogens can quickly degrade grape quality (Guerra and Steenwerth, 2012).

### 3.5 Soil characteristics and their impact on Riesling vine growth

The Southwest Desert’s hot, arid climate imposes significant soil-related constraints on viticulture, directly affecting water availability, nutrient cycling, and overall vine health. Elevated temperatures accelerate organic matter decomposition, leading to a reduction in soil carbon stocks and microbial activity, both of which are critical for maintaining soil fertility and resilience (Alam et al., 2022). Simultaneously, limited annual rainfall combined with high evaporation rates promotes the accumulation of salts in the root zone, impairing nutrient uptake, degrading soil structure, and ultimately stressing grapevines (Mpanga et al., 2024). Soil water retention is a major challenge in this region; while sandy soils drain rapidly and require frequent irrigation, certain areas with heavy clay soils can also present issues, such as poor drainage and waterlogging during rare precipitation events (Marín-Martínez et al., 2021). Additionally, the formation of hard surface crusts on dry, sandy soils restricts root penetration and limits water infiltration, further exacerbating vine water stress. Together, these soil challenges necessitate careful vineyard site selection, soil amendment strategies, and highly efficient irrigation management to support sustainable viticulture under desert conditions.

## 4 Mitigation strategies for climate variability

To address extreme weather in the Desert Southwest, the literature suggests targeted adaptation strategies suited to arid and variable conditions, such as heat control, soil management, irrigation techniques, site selection, and agronomical practices.

### 4.1 Soil management strategies: mulching, cover crops, and soil amendments

Effective soil and water management is essential for successfully cultivating Riesling grapes in desert environments. A combination of techniques, including mulching, cover cropping, and targeted soil amendments, plays a synergistic role in enhancing soil structure, conserving water, and moderating temperature extremes.

Mulching, whether organic or synthetic, serves as an effective strategy to mitigate the harsh soil microclimatic conditions. In high-radiation environments where daytime soil surface temperatures can exceed 50°C, mulching plays a pivotal role in reducing soil temperature by 5–10°C, thereby protecting vine root systems from thermal stress and preserving beneficial soil microbial communities (Mairata et al., 2024; Cabrera-Pérez et al., 2023).

One of the primary benefits of mulching is its ability to minimize soil water loss through evaporation. By creating a physical barrier between the soil and the atmosphere, mulches reduce the direct impact of solar radiation and wind, significantly lowering evapotranspiration rates and contributing to improved water-use efficiency (WUE) (Intrigliolo and Castel, 2010). In addition to enhancing moisture retention, mulching also stabilizes diurnal temperature fluctuations at the soil surface, providing a more favorable and consistent root-zone environment for vine growth. Beyond thermal and hydrological regulation, mulching contributes to weed suppression, erosion control, and soil structure preservation (Costa et al., 2023). Organic mulches offer the added benefit of slowly decomposing and contributing to soil organic matter, which can enhance nutrient cycling and microbial activity (Mairata et al., 2024). These multifaceted benefits make mulching a cornerstone practice for sustainable viticulture in arid and semi-arid regions, particularly for sensitive cultivars like Riesling that require stable root-zone conditions for optimal physiological performance.

Cover cropping, the intentional cultivation of non-cash crops such as legumes (e.g., *Vicia villosa*) or grasses (e.g., *Festuca* spp., *Lolium* spp.) between vine rows, has emerged as a key soil management strategy in arid and semi-arid viticultural regions. Integrating cover crops into vineyard floor management provides a range of agronomic and ecological benefits that directly enhance vine health and productivity.

One of the most critical contributions of cover cropping is the improvement of soil organic matter (SOM) and soil organic carbon (SOC). The incorporation of cover crop residues, along with root biomass turnover, introduces carbon-rich organic materials into the soil, which increases SOC levels over time. Elevated SOC improves soil structure, nutrient-holding capacity, and water infiltration, leading to better root aeration and enhanced moisture availability especially crucial in water-limited environments (Guerra and Steenwerth, 2012).

In addition, cover crops stimulate soil microbial activity and biodiversity by supplying continuous organic inputs and root exudates. These biological interactions are essential for nutrient cycling and support beneficial microbial communities that contribute to vine resilience under abiotic stress conditions. Cover cropping also offers substantial erosion control benefits, especially in sloped or sandy soils prone to wind and water erosion (Marks et al., 2022). The canopy and root systems of cover crops act as a physical barrier against erosive forces, reducing sediment loss and runoff. Studies have documented that vineyards employing cover crops experience significant reductions in erosion (up to 60%) and increases in SOC (15–25%) compared to bare or tilled vineyard rows (Giffard et al., 2022).

To improve the water-holding capacity, nutrient retention, and overall fertility of desert vineyard soils, a combination of organic and mineral amendments is highly effective. Organic materials such as compost, manure, and biochar enhance soil structure, increase soil organic matter (SOM), and improve microbial activity and moisture retention, while also boosting cation exchange capacity (CEC) (Martínez-Lüscher et al., 2020; Jeffery et al., 2011). Among these, biochar stands out for its long-term stability in soil and its ability to retain nutrients and water, making it particularly valuable in arid viticultural systems.

In parallel, clay-rich mineral amendments like zeolite, bentonite, and vermiculite further improve CEC, reduce nutrient leaching, and enhance soil water-holding capacity—all critical properties in the sandy, low-organic soils typical of desert regions (Cicchelli et al., 2016; Bigelow et al., 2001). Zeolite and bentonite have been shown to support long-term soil fertility by slowly releasing retained nutrients. When applied in combination, organic and mineral amendments offer synergistic benefits, contributing to sustainable soil health, greater vine resilience, and reduced irrigation frequency a key advantage in water-limited environments (Cataldo et al., 2024). These strategies are especially valuable for the successful cultivation of water-sensitive grape varieties like Riesling in arid viticultural regions.

### 4.2 Canopy management techniques

One of the most effective strategies for regulating fruit zone microclimates in vineyards is canopy management. By modifying vine training systems such as Vertical Shoot Positioning (VSP), Scott Henry, or lyre systems, growers can strategically adjust leaf placement to provide natural shading over grape clusters while maintaining adequate airflow (Reta et al., 2025). These systems help create a balance between sun exposure and protection, which is particularly important in hot and arid growing regions.

Properly managed canopies refer to vine canopy structures that are intentionally trained and maintained through practices such as shoot positioning, leaf removal, hedging, and strategic pruning to optimize light penetration, air circulation, and shading of grape clusters. When implemented effectively, these practices can reduce fruit zone temperatures by up to 10°C during peak afternoon heat, significantly lowering the risk of sunburn, dehydration, and heat-induced berry damage (Costa et al., 2019). In hot climates, additional strategies such as late pruning, delaying winter or early spring pruning, can postpone phenological stages, helping to avoid early-season heat stress and align ripening with cooler periods, thereby preserving acidity and aromatic quality in sensitive cultivars like Riesling (Frioni et al., 2017; Rogiers et al., 2022). Combined, these canopy interventions enhance fruit quality and resilience under desert viticulture conditions.

This cooling effect is vital for maintaining fruit quality, especially in heat-sensitive varieties like Riesling. Additionally, orienting vineyard rows east to west has been shown to enhance the shading effect provided by the canopy. This orientation shields grape clusters from intense afternoon sunlight, particularly in desert climates where solar radiation peaks during the late day hours (Blanco-Ulate et al., 2020). By minimizing direct sun exposure, this approach contributes to better temperature regulation and reduces the risk of heat-related stress on the vine and fruit.

### 4.3 Artificial shading systems

In regions where natural canopy shading is inadequate, especially during prolonged heatwaves, the implementation of artificial shading techniques such as shade cloths or nets has emerged as a valuable tool for protecting grape clusters. These structures are designed to reduce light intensity, moderate fruit zone temperatures, and safeguard overall grape quality.

Shading nets, particularly photoselective and neutral-density polyethylene (PE) nets, are increasingly used in viticulture to mitigate the impact of excessive solar radiation and heat stress in arid and semi-arid regions. These nets typically vary in color (e.g., black, red, white, or green) and shade factor, with 30–50% light reduction being the most applied for grape production (Abad et al., 2023). Photo selective nets modify not only light intensity but also light quality by filtering specific wavelengths, potentially influencing grape metabolism, while neutral-density nets primarily reduce the overall quantity of incident light without altering spectral composition (Caroline et al., 2017).

Research has shown that shade nets can delay the onset of ripening, moderate sugar accumulation, and preserve titratable acidity, making them particularly valuable for heat-sensitive cultivars like Riesling (Zha et al., 2022). For example, a recent field study demonstrated that vineyards using 40% shade nets had significantly better fruit integrity, higher acidity, and lower incidence of sunburn and shrivel compared to unshaded controls (Abad et al., 2023). Beyond physiological benefits, artificial shading also helps preserve varietal typicity by maintaining aromatic compounds and delaying overripe flavor development (Scafidi et al., 2013). As such, shading nets represent a promising adaptation tool in desert viticulture systems aiming to balance ripening kinetics, wine freshness, and fruit quality under increasingly hot growing seasons (Bernardo et al., 2018). These benefits underscore the value of integrating shading into vineyard heat stress management protocols, especially under climate change conditions that are increasing the frequency and intensity of extreme heat events (Martínez-Lüscher et al., 2020).

### 4.4 Kaolin clay and reflective sprays

An increasingly popular, non-invasive strategy for mitigating heat stress in vineyards is the application of kaolin clay and other reflective particle films to vine canopies. These materials form a thin, white, reflective barrier on leaves and fruit, significantly decreasing the absorption of solar radiation and lowering tissue temperatures without negatively impacting photosynthesis. Studies have demonstrated that kaolin application can reduce berry surface temperatures by approximately 5–7°C and decrease sunburn incidence by over 40% compared to untreated vines, offering crucial protection during periods of intense solar radiation (Teker, 2023). Kaolin-based reflective coatings are typically applied starting at fruit set and reapplied every 2–4 weeks through veraison to pre-harvest, particularly during periods of intense heat and solar radiation (Glenn and Puterka, 2010; Dinis et al., 2018). The white film reflects sunlight, reducing canopy and berry temperatures, and improving water use efficiency by lowering stomatal conductance and transpiration. Qualitatively, kaolin helps delay ripening, preserve acidity, and moderate sugar accumulation, which supports better balance and freshness in heat-sensitive cultivars like Riesling (Petoumenou, 2023). Combined with shade nets, kaolin amplifies microclimate regulation, reduces sunburn and dehydration, and enhances fruit integrity and aromatic preservation under heat stress (Zha et al., 2022).

Given the challenges posed by climate change, particularly in arid and semi-arid viticultural regions, the integration of reflective materials like kaolin into vineyard management practices represents a promising, low-cost adaptation strategy for preserving both vine health and grape quality.

### 4.5 Vine elevation and trellising systems to mitigate temperature stress

Elevating grapevines through carefully selected trellising systems is a critical strategy for mitigating heat stress, particularly in desert vineyard regions. When the grapevine is trained higher off the ground, the canopy is exposed to greater airflow, which helps lower ambient temperature around the fruit zone and enhances evapotranspiration cooling.

By elevating vines above the soil surface, the reflected heat can be reduced, which in turn limits berry sunburn and dehydration factors, especially detrimental to delicate cultivars like Riesling (Reta et al., 2025; Blanco-Ulate et al., 2020).

Trellising systems such as vertical shoot positioning (VSP), Smart-Dyson, lyre, and open gable are widely used to manipulate canopy geometry, optimize sunlight interception, and promote uniform ripening. For example, high-wire cordon systems maintained lower fruit zone temperatures and delayed ripening, helping preserve acidity and aromatic compounds in Riesling grape varieties (Reynolds et al., 1996; Danko et al., 2024). Elevated vine canopies improve access for machinery used in mechanical harvesting, canopy management, and spraying, which reduces labor costs and improves consistency, important considerations for commercial vineyards in arid zones (Garcia et al., 2022).

Raising the fruiting wire height in vineyards can help reduce frost risk by positioning buds above the coldest air near the ground. Cold air settles close to the soil, increasing the likelihood of frost damage to low-positioned shoots. Research indicates that vines trained at 1.4 meters can be up to 0.5°C warmer than those at 0.9 meters (Terpou et al., 2024). A height between 0.9–1.2 meters is generally recommended to balance frost protection with ease of vineyard operations (Chien, 2013) In high-risk areas, some growers adopt high cordon systems above 1.5 meters (McCloskey et al., 2023), though this may require more intensive canopy and labor management.

### 4.6 Selecting drought-tolerant rootstocks for arid viticulture

Selecting appropriate drought-tolerant rootstocks can help sustain grapevine performance under water-limited conditions. This strategy is particularly applicable for Riesling, which tends to have shallow root systems and is sensitive to drought and nutrient imbalances. The right rootstock not only enhances drought resistance but also improves nutrient uptake in high-pH, low-organic-matter soils commonly found in desert viticulture. [Note here that rootstocks can be problematic if they die back due to frost damage. In that case, the vine will have to be replaced. If it is own-rooted, the vine can be left in place to regrow from the root, even though there is a significant setback in production.]

When cultivating *Riesling* grapes in arid and semi-arid regions such as Arizona, southern California, and New Mexico, selecting an appropriate rootstock can help manage water scarcity and ensure vine health. Few Rootstocks have been studied for their performance under water-deficit conditions.​Several commercially available rootstocks have been identified for their adaptability to drought and poor soil conditions. Among the most effective are:

- Ramsey (*Vitis champinii*): Known for its vigorous growth and deep root system, Ramsey exhibits high drought tolerance. Studies have shown that Ramsey maintains higher stomatal conductance and photosynthesis rates under moderate water deficit compared to other rootstocks, indicating its efficiency in water uptake and utilization (Cochetel et al., 2020).
- 1616 Couderc (1616C): This rootstock is characterized by its moderate vigor and good resistance to certain soil pests. However, its performance under drought conditions is less favorable compared to more drought-tolerant rootstocks. 1616C tends to have a shallower root system, which may limit its ability to access deep soil moisture during prolonged dry periods. Therefore, while 1616C may be suitable for regions with adequate irrigation or higher rainfall, it may not be the optimal choice for arid climates without supplemental water sources
- 110 Richter (110R) – A hybrid of *Vitis berlandieri* × *V. rupestris*, known for its vigorous growth and excellent drought tolerance. Its deep root system allows access to subsurface moisture, making it suitable for dry-farmed or deficit-irrigated vineyards (IFV, 2023a).
- 1103 Paulsen (1103P) – Another *berlandieri* × *rupestris* cross, with strong resistance to lime-induced chlorosis and good tolerance to salinity and high-pH soils. It performs well in calcareous, low-fertility soils typical of desert regions (IFV, 2023b).
- 140 Ruggeri (140Ru) – This rootstock has one of the deepest rooting profiles and is highly resistant to drought, salinity, and nematodes. It is especially beneficial in sandy and arid soils where water retention is poor (Tramontini et al., 2013; Ollat et al., 2015).

Riesling is compatible with drought-tolerant rootstocks like *110R*, *1103P*, and *140Ru*, which improve water uptake and help preserve acidity under heat stress—ideal for arid climates (Ollat et al., 2015; Nader et al., 2019). In contrast, *SO4* and *5BB* are less drought-resistant but may be suitable with irrigation for earlier ripening (Tramontini et al., 2013). Rootstock choice should balance drought tolerance and fruit quality.

The structure and function of grapevine roots are key to surviving dry conditions. These include roots that shrink less during dry periods, so they stay in contact with the soil; better water uptake through more efficient water channels; and strong communication between roots and shoots. These features help vines stay healthier and use water more efficiently during drought (Pech et al., 2013; Li et al., 2021). Researchers revealed that specific traits such as xylem anatomy and reduced embolism formation under water stress were strongly linked to the drought tolerance of rootstocks like 1103P and 140Ru, making them ideal for dry regions like Arizona, southern California, and New Mexico (Li et al., 2021). Moreover, drought-tolerant rootstocks contribute to long-term sustainability, enabling growers to reduce irrigation frequency and manage vineyards more efficiently amid tightening water regulations and prolonged drought cycles.

### 4.7 Microbial inoculation as a sustainable strategy to improve soil health

Desert soils often lack organic matter and essential nutrients, making soil improvement a crucial component of vineyard management. Adding organic matter such as compost, cover crops, or manure improves soil fertility, microbial activity, and water retention (Blanco et al., 2017). Many desert soils are alkaline, which affects nutrient availability; applying sulfur or organic acids can balance soil pH and enhance vine nutrient uptake (Martínez-Lüscher et al., 2020).

**Figure 5:**
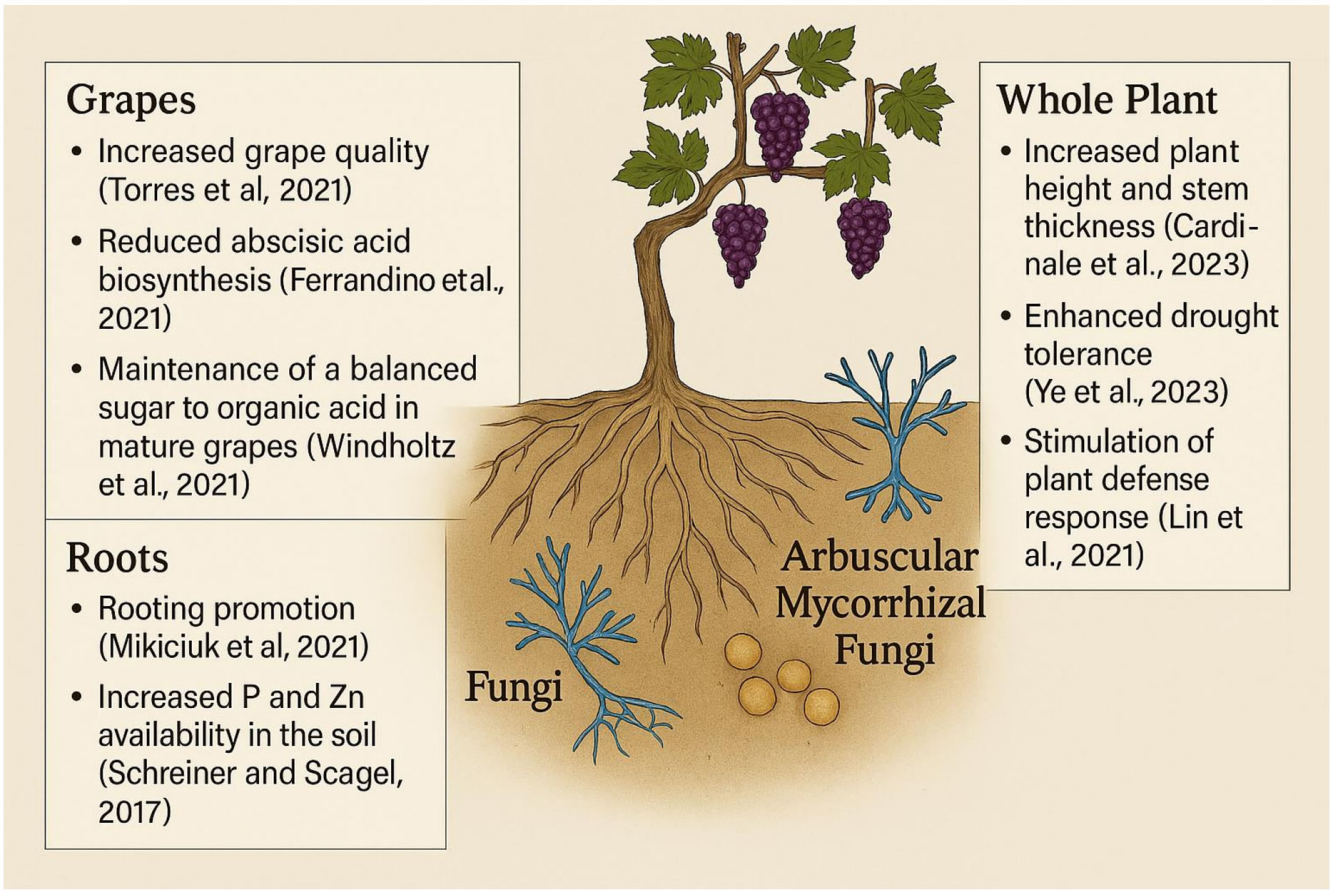
Effect of microbial inoculation of grapevine.

Introducing beneficial soil microbes, such as nitrogen-fixing rhizobacteria and mycorrhizal fungi, can significantly enhance nutrient availability and support the development of robust root systems adapted to challenging conditions (**Figure 5**) (Aguilera et al., 2022). These microbial partnerships not only improve the soil’s biological activity but also increase water-use efficiency critically in desert viticulture (Aguilera et al., 2022; Kozikova et al., 2024). As a result, growers can achieve healthier vines, improved grape composition, and a more sustainable approach to vineyard management (Khalil, 2012; Samantaray et al., 2024). Microbial inoculation, involving beneficial soil microbes and mycorrhizal fungi, offers a sustainable alternative to synthetic fertilizers by enhancing nutrient uptake, improving root development, and supporting overall soil health (Aguilera et al., 2022). In nutrient-poor vineyard soils, especially those in arid regions, this approach can improve vine productivity, grape composition, and environmental sustainability (Khalil, 2012; Samantaray et al., 2024).

### 4.8 Integrating technology into vineyard culture

#### 4.8.1 Agrivoltaics

**Figure 6:**
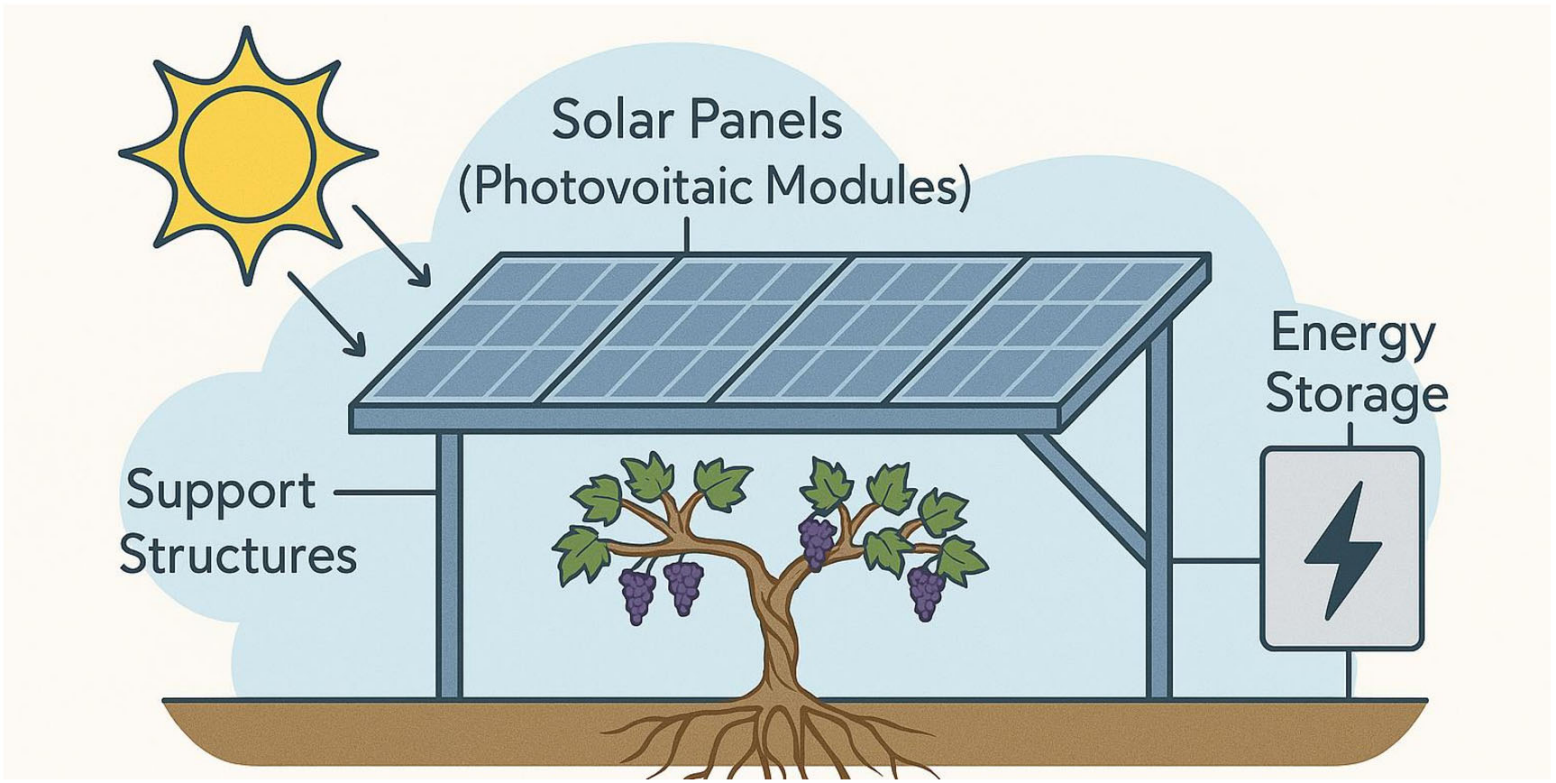
Agrivoltaics system for sustainable viticulture: integrating solar energy with grape production.

Integrating agrivoltaics into vineyard culture, defined as the simultaneous use of land for photovoltaic energy generation and agriculture, has emerged as a climate-smart strategy with significant potential for viticulture (**Figure 6**) (Magarelli et al., 2025; Walston et al., 2022). Increasing vulnerability of vineyards to climate extremes, including heatwaves, drought, and erratic precipitation, the integration of solar panels into vineyard systems offers a multifaceted solution to enhance both environmental and economic resilience (Magarelli et al., 2025; Weselek et al., 2019). Agrivoltaic installations can create beneficial microclimates by moderating solar radiation, lowering canopy and soil temperatures, and reducing evapotranspiration, thereby improving water-use efficiency and protecting grape quality (Magarelli et al., 2025; Fumey et al., 2023).

Recent research has demonstrated that elevated and semi-transparent solar arrays can be effectively integrated into vineyard architecture, maintaining adequate light transmission for photosynthesis while providing shading benefits during critical phenological stages (Weselek et al., 2019). Riesling maintains optimal photosynthesis at light transmittance levels of 30–35%, making it suitable for semi-transparent agrivoltaic systems. Pilot study show that panels allowing 32–40% light transmission can support adequate CO_2_ assimilation while reducing heat stress. However, performance may decline if transmittance falls below 25%, depending on canopy density and site conditions.

Although agrivoltaic systems yield 10–20% less energy than conventional solar farms, this is offset by benefits such as reduced crop damage, lower irrigation needs, improved grape quality, and on-site energy use. Models show that, when accounting for both energy and crop value, net returns per hectare can exceed those of standalone viticulture or solar, especially for high-value cultivars like Riesling (Heinitz et al., 2020). Furthermore, the adoption of agrivoltaics aligns with growing market trends toward sustainable and carbon-neutral wine production. Nonetheless, challenges remain, including high initial investment costs, regulatory barriers, and potential aesthetic concerns in culturally sensitive wine regions. Feasibility studies, including the Eureka County Agrivoltaics Study (https://www.nature.org/en-us/newsroom/nevada-agrivoltaics-feasibility-study/, 2022) demonstrate the potential of agrivoltaics in U.S. desert viticulture by highlighting its dual benefits: renewable energy production and enhanced crop resilience. While high initial costs and regulatory barriers remain, agrivoltaics is emerging as a practical solution for climate-adaptive, energy-efficient wine production, with growing interest in pilot projects across Europe and North America (Adeh et al., 2021; Fumey et al., 2023). **Table 4** shows the potential use of agrivoltaics in the vineyard culture.

**Table 4:** Summary of agrivoltaics integration into vineyard culture.

Aspect	Description/Insights	Reference(s)
System Design	Elevated fixed or tracking solar PV panels; often aligned with vineyard rows or trellis structures.	Weselek et al., 2019; Fumey et al., 2023
Microclimate Regulation	Reduces canopy temperature and solar stress; preserves berry acidity; improves water retention and reduces evapotranspiration.	Magarelli et al., 2025
Impact on Grapevines	Slight delay in ripening; improved sugar-acid balance in hot climates; moderate shading does not harm yield in tolerant varieties.	Magarelli et al., 2025; Marrou et al., 2013
Energy Generation	On-site solar energy for irrigation, cooling, and processing; potential to sell excess energy to grid.	Heinitz et al., 2020; Adeh et al., 2021
Water Use Efficiency	Shading leads to lower soil water loss, reducing irrigation frequency and enhancing resilience in drought-prone areas.	Marrou et al., 2013
Economic Benefits	Dual income from crop + energy; reduced input costs (water, electricity); enhanced sustainability branding for wine.	Heinitz et al., 2020
Challenges	High initial cost; changes to vineyard aesthetics; potential regulatory and zoning constraints; maintenance around panels.	Fumey et al., 2023; Adeh et al., 2021
Social Acceptance	May face resistance in traditional wine regions; however, aligns with eco-certification and consumer demand for sustainable products.	Weselek et al., 2019
Current Applications	Pilot projects in France, Germany, Italy, South Korea, and U.S. (e.g., California and Oregon); research ongoing in Mediterranean and arid climates.	Fumey et al., 2023

#### 4.8.2 Application of AI and IoT in vineyards

The integration of Artificial Intelligence (AI) and the Internet of Things (IoT) in vineyard management refers to the use of smart sensors, real-time data collection, and machine learning algorithms to monitor and control key vineyard conditions. For Riesling cultivation in arid regions, this approach enables precise optimization of irrigation schedules, targeted nutrient delivery, and adaptive responses to climate stress, enhancing grape quality while conserving resources (**Table 5**). AI models and IoT sensors monitor environmental variables like soil moisture, temperature, and plant stress, enabling precise, data-driven irrigation management that enhances water efficiency and grape quality (Wolfert et al., 2017). IoT-based irrigation systems have shown promising results in the Desert Southwest, with trials in Arizona and California reporting 20–40% water savings and improved grape quality. While most validations have been plot-scale, full-season and commercial-scale studies are expanding, with systems increasingly tailored to real-time VPD and soil moisture data for precision irrigation under arid conditions (García et al., 2020). Machine learning further refines irrigation scheduling by analyzing historical climate data to predict drought conditions and optimize water use (Shamshiri et al., 2018).

**Table 5:** Applications of AI and IoT in vineyard management.

Technology	Application	Benefits	Example tools	Reference
AI	Disease prediction	Early detection and prevention	VineView, PlantVillage	Izquierdo-Bueno et al., 2024
AI	Yield forecasting	Helps in harvest planning and logistics	IBM Watson Decision Platform	
AI	Image analysis for grape quality	Non-destructive ripeness assessment	Computer Vision (e.g., CNNs)	Izquierdo-Bueno et al., 2024
IoT	Soil moisture monitoring	Precision irrigation, water conservation	Arable, Sentek, AquaSpy	García et al., 2020
IoT	Microclimate monitoring	Fungus risk alerts, frost warnings	Davis Vantage Pro2, Sencrop	Chedea et al., 2021
IoT	Autonomous irrigation control	Reduces manual labor, maximizes water use	CropX, Netafim	García et al., 2020
AI + IoT	Integrated vineyard management platforms	Unified dashboard for real-time data decisions	VineSense, Viticulture Data Hub	Izquierdo-Bueno et al., 2024; Chedea et al., 2021

AI-driven nutrient management improves soil fertility by using sensors to monitor nutrient levels and microbial activity, providing data for precise fertilization tailored to vine needs (Liakos et al., 2018). Remote sensing technologies such as drones and satellite imagery enhance vineyard monitoring, enabling early detection of plant stress, pests, and diseases, thus reducing chemical interventions and promoting sustainability (Maes and Steppe, 2019). Climate modeling plays a crucial role in identifying suitable microclimates for Riesling. AI-powered models analyze historical weather patterns and predict future climate conditions to guide vineyard placement and adaptation strategies (Droulia and Charalampopoulos, 2021). Techniques like windbreaks, reflective mulches, and shade nets help mitigate climate variability, while real-time IoT weather stations improve yield forecasting and harvest timing (Mirás-Avalos and Araujo, 2021).

## 5 Conclusion

Growing Riesling in the desert Southwest of the United States presents significant challenges due to extreme heat, water scarcity, and poor soil conditions, all of which compromise vine growth, fruit development, and the preservation of the cultivar’s hallmark acidity and aromatic profile. Despite these limitations, this review highlights the feasibility of cultivating Riesling in arid environments through an integrated adaptation strategy. Key viticultural interventions include high-elevation site selection, drought-tolerant rootstocks, and canopy or trellising systems that reduce heat stress and sunburn. Soil health is supported through mulching, compost application, and microbial inoculation, enhancing water retention and nutrient availability. Complementing these practices are emerging technologies such as IoT-enabled smart irrigation, AI-based climate modeling, and remote sensing, which enable more precise and adaptive vineyard management. Of note is the application of agrivoltaics—solar panels installed above vine rows that provide partial shading while generating renewable energy, simultaneously mitigating heat load and reducing water demand.

The novelty of this review lies in its multidisciplinary perspective, integrating traditional viticulture with precision agriculture and renewable energy technologies to adapt a cool-climate cultivar to extreme desert conditions. Future research should focus on optimizing rootstock-scion combinations, evaluating shading systems (e.g., nets vs. agrivoltaics), and assessing long-term impacts on wine composition and sensory typicity to ensure the viability of high-quality Riesling production in these emerging terroirs.
