# Correction: Challenges and adaptation strategies for Riesling grape (*Vitis vinifera* L) production in the southwest desert in the USA

**DOI:** 10.3389/fpls.2025.1709314

**Published:** 2025-10-06

**Authors:** Most Tahera Naznin, Md Obyedul Kalam Azad, Jill Moe

**Affiliations:** ^1^ Department of Agriculture, Veterinary and Rangeland Sciences, Nevada Agricultural Experiment Station, University of Nevada, Reno, Las Vegas, NV, United States; ^2^ Desert Farming Initiative, University of Nevada, Reno, NV, United States

**Keywords:** Riesling cultivation, desert viticulture, water management, climate adaptation, precision agriculture

“Department of Agriculture, Veterinary and Rangeland Sciences, Nevada Agricultural Experiment Station, University of Nevada, Reno, Las Vegas, NV, United States” was erroneously given “^1^Department of Agriculture, Veterinary and Rangeland Sciences, Nevada Agricultural Experiment Station, University of Nevada, Las Vegas, NV, United States”

The original version of this article has been updated.

